# Life at Home and on the Roam: Genomic Adaptions Reflect the Dual Lifestyle of an Intracellular, Facultative Symbiont

**DOI:** 10.1128/mSystems.00057-19

**Published:** 2019-05-07

**Authors:** Ilia Burgsdorf, Kim M. Handley, Rinat Bar-Shalom, Patrick M. Erwin, Laura Steindler

**Affiliations:** a Department of Marine Biology, Leon H. Charney School of Marine Sciences, University of Haifa, Haifa, Israel; b School of Biological Sciences, The University of Auckland, Auckland, New Zealand; c Department of Biology and Marine Biology, Centre for Marine Science, University of North Carolina—Wilmington, Wilmington, North Carolina, USA; Vanderbilt University

**Keywords:** *Petrosia ficiformis*, *Synechococcus feldmannii*, *Synechococcus spongiarum*, comparative genomics, endosymbionts, intracellular bacteria, metagenome assembled genomes, sponge, symbiosis

## Abstract

“*Candidatus* Synechococcus feldmannii” is a facultative intracellular symbiont of the Atlanto-Mediterranean sponge Petrosia ficiformis. Genomic information of sponge-associated cyanobacteria derives thus far from the obligate and extracellular symbiont “*Candidatus* Synechococcus spongiarum.” Here we utilized a differential methylation-based approach for bacterial DNA enrichment combined with metagenomics to obtain the first draft genomes of “*Ca.* Synechococcus feldmannii.” By comparative genomics, we revealed that some genomic features (e.g., iron transport mediated by siderophores, eukaryotic-like proteins, and defense mechanisms, like CRISPR-Cas [clustered regularly interspaced short palindromic repeats-associated proteins]) are unique to both symbiont types and absent or rare in the genomes of taxonomically related free-living cyanobacteria. These genomic features likely enable life under the conditions found inside the sponge host. Interestingly, there are many genomic features that are shared by “*Ca*. Synechococcus feldmannii” and free-living cyanobacteria, while they are absent in the obligate symbiont “*Ca.* Synechococcus spongiarum.” These include genes related to cell surface structures, genetic regulation, and responses to environmental stress, as well as the composition of photosynthetic genes and DNA metabolism. We speculate that the presence of these genes confers on “*Ca.* Synechococcus feldmannii” its facultative nature (i.e., the ability to respond to a less stable environment when free-living). Our comparative analysis revealed that distinct genomic features depend on the nature of the symbiotic interaction: facultative and intracellular versus obligate and extracellular.

**IMPORTANCE** Given the evolutionary position of sponges as one of the earliest phyla to depart from the metazoan stem lineage, studies on their distinct and exceptionally diverse microbial communities should yield a better understanding of the origin of animal-bacterium interactions. While genomes of several extracellular sponge symbionts have been published, the intracellular symbionts have, so far, been elusive. Here we compare the genomes of two unicellular cyanobacterial sponge symbionts that share an ancestor but followed different evolutionary paths—one became intracellular and the other extracellular. Counterintuitively, the intracellular cyanobacteria are facultative, while the extracellular ones are obligate. By sequencing the genomes of the intracellular cyanobacteria and comparing them to the genomes of the extracellular symbionts and related free-living cyanobacteria, we show how three different cyanobacterial lifestyles are reflected by adaptive genomic features.

## Abstract (precis)

Given the evolutionary position of sponges as one of the earliest phyla to depart from the metazoan stem lineage, studies on their distinct and exceptionally diverse microbial communities should yield a better understanding of the origin of animal-bacterium interactions. While genomes of several extracellular sponge symbionts have been published, the intracellular symbionts have, so far, been elusive. Here we compare the genomes of two unicellular cyanobacterial sponge symbionts that share an ancestor but followed different evolutionary paths—one became intracellular and the other extracellular. Counterintuitively, the intracellular cyanobacteria are facultative, while the extracellular ones are obligate. By sequencing the genomes of the intracellular cyanobacteria and comparing them to the genomes of the extracellular symbionts and related free-living cyanobacteria, we show how three different cyanobacterial lifestyles are reflected by adaptive genomic features.

## INTRODUCTION

Sponges are considered one of the earliest-branching multicellular animals (Metazoa) (1, 2). They inhabit marine and freshwater environments (3–5) and form intimate symbiotic interactions with complex communities of more than 60 phyla of bacteria, including cyanobacteria (6). The latter are a very diverse group with enormous influence on many processes at global scales, due to their ability to perform oxygenic photosynthesis (7–9). The two major groups of cyanobacteria have pronounced differences in their taxonomic affiliations, genome sizes and contents, morphological phenotypes, and developmental capacities of the cell (10–14). The first group, clade 1 (10), includes the highly diverse filamentous cyanobacteria that have dominated marine and freshwater benthic environments for more than 2,300 million years (11). The second group, clade 2 (10), includes marine unicellular cyanobacteria that originated much later (1,000 to 542 million years ago [Mya]) (11) and contributed to the emergence of metazoans, including the early-branching sponges (15), following the “Neoproterozoic oxygenation event” (11, 16–20). Modern planktonic cyanobacteria related to clade 2 consist of the recently proposed genus *Parasynechoccocus* (12–14) and its sister clade, *Prochlorococcus*.

Sponge-associated cyanobacteria are polyphyletic, implying that they derived from multiple independent symbiotic events (21, 22). Unicellular cyanobacteria are the most commonly reported and are widespread in sponges (21), in particular “*Candidatus* Synechococcus spongiarum,” which consists of at least 12 different clades and is found in many different sponge species (23). A less common cyanobacterial symbiont, first defined as Aphanocapsa feldmannii (24) and later named “*Candidatus* Synechococcus feldmannii” (25), is a symbiont in the sponge species Petrosia ficiformis (Poiret, 1789) (21), which also harbors a dense and diverse microbiome (26). One study also reported the presence of “*Ca.* Synechococcus feldmannii” in Ircinia variabilis; however, the identification was based solely on morphology (24). Sequencing of the 16S-23S rRNA internal transcribed spacer regions from six *I. variabilis* specimens from Spain resulted in the identification of “*Ca.* Synechococcus spongiarum” and not “*Ca.* Synechococcus feldmannii” (27).

While “*Ca.* Synechococcus spongiarum,” like most sponge symbionts, is extracellular and vertically transmitted (via “leaky” vertical transmission) (28–31), “*Ca.* Synechococcus feldmannii” is thought to be transferred horizontally, as sponge oocytes of *P. ficiformis* were shown to lack symbiotic bacteria (32). In general, environmental acquisition of symbionts in *P. ficiformis* is supported by known biogeographic influences on the composition of its sponge-associated microbial community (26). Unlike “*Ca.* Synechococcus spongiarum,” “*Ca.* Synechococcus feldmannii” is localized inside specialized host cells called bacteriocytes (32, 33). The scenario of a facultative and horizontally transmitted intracellular symbiont (“*Ca.* Synechococcus feldmannii”) and an obligate and vertically transmitted extracellular symbiont (“*Ca.* Synechococcus spongiarum”) is peculiar. In other symbioses (e.g., insects with bacteria), symbionts present in bacteriocytes are usually linked to obligate relationships, where the symbiont relies on host-based mechanisms for transmission (34). In contrast, the obligate and vertically transmitted cyanobacterial symbiont “*Ca.* Synechococcus spongiarum” is found extracellularly in the sponge host rather than in bacteriocytes. Extracellular, obligate, and vertically transmitted symbionts can be also found in insects (35), yet they are understudied compared to their intracellular counterparts. While genome-based studies of extracellular and (primarily) vertically transmitted sponge symbionts have occupied the focus of published studies, including the cyanobacterium “*Ca.* Synechococcus spongiarum” (36, 37), genomes of bacteriocyte-associated sponge symbionts have so far been neglected. The characterization of facultative, intracellular sponge symbiont genomes will contribute to our understanding of the impact of endo-cellularity and transmission mode on bacterial genome evolution.

Here, we report the first genomes for the facultative intracellular species “*Ca.* Synechococcus feldmannii.” We compare these genomes with those from the extracellular, obligate symbiont species “*Ca.* Synechococcus spongiarum” and with free-living cyanobacterial counterparts to reveal evidence of genomic adaptations to the different symbiotic lifestyles.

## RESULTS

### Genome recovery.

Three draft genomes of “*Ca.* Synechococcus feldmannii” (277cV, 277cI, and 288cV) were obtained from two specimens of the Mediterranean sponge *P. ficiformis* (277 and 288). The pangenome and core genome of the three “*Ca*. Synechococcus feldmannii” specimens consisted of 2,924 genes and 1,338 genes, respectively. The core genome of the three “*Ca*. Synechococcus feldmannii” and six “*Ca*. Synechococcus spongiarum” specimens consisted of only 353 genes. “*Ca.* Synechococcus feldmannii” possesses relatively high GC content and small genome size similar to “*Ca.* Synechococcus spongiarum” and members of *Parasynechoccus* (36). Additional information on the “*Ca*. Synechococcus feldmannii” genomes is provided in Table 1.

**TABLE 1:** Genomic information for the three “*Ca.* Synechococcus feldmannii” assemblies 277cV, 277cI, and 288cV

Assembly	Result for assembly		
	277cV	277cI	288cV
Genome size (Mb)	2.5	1.9	2.2
Avg GC content (%)	62.9	64.7	64
Completeness (%)	94.1	86.1	87.8
Contamination (%)	0.0	0.3	0.0
Heterogeneity (%)	0.0	0.0	0.0
No. of:			
Scaffolds	100	214	125
ORFs (Prodigal)	2,506	2,137	2,158
COGs^*a*^	1,084	1,018	1,008
SEED functions^*a*^	856	810	811
ORFs (RAST)	2,349	1,942	1,907
Ratio to pangenome (%)	80.3	66.4	65.2
No. of SNPs^*b*^	4,467	1,980	11,826
Nonsynonymous SNPs (%)^*b*^	73.5	66.5	77.5
Genes with SNPs (%)^*b*^	14.1	17.6	82.5
No. of scaffolds with SNPs^*b*^	100 (all)	210 (out of 214)	125 (all)
No. of SNPs^*c*^	1,349 for 277cI, 8,789 for 288cV		6,301 for 277cI
Nonsynonymous SNPs (%)^*c*^	73.7 for 277cI, 78.6 for 288cV		77.8 for 277cI

a Total number of unique COG and SEED annotations.

b Illumina reads were mapped to the assembly.

c Artificial reads produced from another genome were mapped to the assembly. The source of the artificial reads is given.

### Intraspecific genomic diversity.

The three genomes of “*Ca.* Synechococcus feldmannii” showed similar gene architectures for homologous regions. However, each assembly contained different gene composition and single-nucleotide polymorphism (SNP) variations. A total of 1,349 SNPs were detected between the 277cV and 277cI genomes derived from the same sponge specimen (Table 1). The number of SNPs between the 277cV and 288cV, which was derived from a different *P. ficiformis* specimen, was higher: 8,789. Mapping of raw Illumina reads to the 277cV and 277cI genomes showed lower intragenomic variability (14.1% and 17.6% of genes had SNPs) than the 288cV genome (82.5%). However, the percentages of nonsynonymous mutations related to SNPs within the coding regions were relatively constant, ranging between 66.5% and 77.5% (Table 1). The genes containing SNPs within genome 277cV included all genes harboring fibronectin type III (FN3) and ankyrin (ANK) domains, 13 out of 14 genes coding for proteins with the leucine-rich repeat (LRR) domains, and 9 out of 10 CRISPR-Cas (clustered regularly interspaced short palindromic repeats-associated proteins)-related genes in the genome 277cI.

### “*Ca.* Synechococcus feldmannii” and “*Ca.* Synechococcus spongiarum” are *Parasynechococcus*-like sponge-associated cyanobacteria.

**FIG 1:**
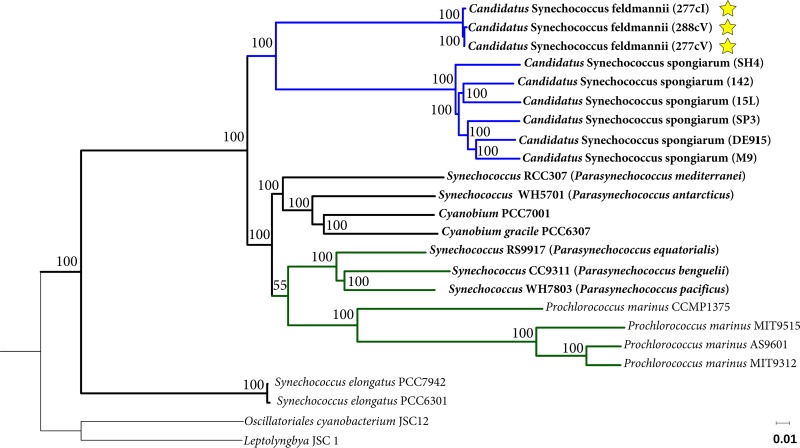
Concatenated phylogenetic core genome tree calculated by iterative pairwise comparison of genomes of the cyanobacteria analyzed here. Bootstrap values at branch nodes derive from 500 replications. The names of the here analyzed genomes are represented in bold. The genomes of “*Ca*. Synechococcus feldmannii” are marked with a star. Symbiotic cyanobacteria and the marine *Parasynechococcus*/*Prochlorococcus* subclade are marked with the blue and green branches, respectively. Cyanobacteria belonging to clade 2 (10) are marked with bold branches. *Oscillatoriales cyanobacterium* JSC12 and *Leptolyngbya* sp. strain JSC 1 belonging to clade 1 (10) were used as an outgroup for tree rooting.

According to a phylogenomic analysis of 24 genomes (including 3 genomes of “*Ca.* Synechococcus feldmannii,” 6 genomes of “*Ca.* Synechococcus spongiarum” and 15 genomes of free-living cyanobacteria) based on 135 core genes common to all genomes, “*Ca.* Synechococcus feldmannii” is most closely related to “*Ca.* Synechococcus spongiarum” (Fig. 1). These symbionts, together with *Prochlorococcus* and small-celled and mostly marine unicellular *Synechococcus* and Synechococcus elongatus, belong to cyanobacterial clade 2 and cluster separately from *Synechococcus* within cyanobacterial clade 1, characterized by a larger genome size and diverse phenotype (10). To address this phylogenetic characteristic of the group of closely related unicellular *Synechococcus* species, a proposal has recently been put forward to rename the group *Parasynechococcus* (13, 14). Thus, we refer to the symbionts as *Parasynechococcus*-like sponge-associated cyanobacteria.

### The worldwide distribution of *Parasynechococcus*-like sponge-associated cyanobacteria.

We analyzed the distribution and abundance of “*Ca.* Synechococcus feldmannii” and “*Ca.* Synechococcus spongiarum” using the Sponge Microbiome Project data set (6), which is part of the Earth Microbiome Project (EMP [www.earthmicrobiome.org]) and includes 2,882 sponge samples from 235 sponge species, 308 seawater samples, 54 marine sediment samples, and 1 algal tissue sample, collected from 37 countries. “*Ca.* Synechococcus feldmannii” and “*Ca.* Synechococcus spongiarum” were most closely affiliated (100% identity) to operational taxonomic units (OTUs) OTU0000398 and OTU0000007, respectively. “*Ca.* Synechococcus feldmannii” was found to be significantly enriched only in *P. ficiformis* (M. Britstein, C. Cerrano, L. Zoccarato, I. Burgsdorf, N. J. Kenny, A. Riesgo, M. Lalzar, and L. Steindler, submitted for publication), establishing “*Ca.* Synechococcus feldmannii” as an intracellular symbiont that is highly specific to a single sponge species. In contrast, “*Ca.* Synechococcus spongiarum” was significantly enriched in 28 different sponge species (see Fig. S1 in the supplemental material) sampled in 21 (out of 37) countries around the globe. Nine sponge species significantly enriched in “*Ca.* Synechococcus spongiarum” were previously found to have photosynthetic activity based on measurements of photosynthetic quantum yield by pulse amplitude-modulated (PAM) fluorometry (38). Conversely, all the PAM-negative sponge species lacked significant enrichment in “*Ca.* Synechococcus spongiarum.” Interestingly, “*Ca.* Synechococcus spongiarum” was relatively abundant in three samples of *P. ficiformis* from São Miguel Island, Portugal (1.84 to 5.63%), while the same specimens lacked their typical symbiont, “*Ca.* Synechococcus feldmannii.”

10.1128/mSystems.00057-19.2 FIG S1 Relative abundances of OTUs with 99% identity to the 16S rRNA of “*Ca*. Synechococcus spongiarum” in the Sponge Microbiome Project. Presented in the figure are all sponge species significantly enriched with “*Ca*. Synechococcus spongiarum” (marked with a star) that are positive or negative for photosynthetic activity based on PAM (green and red, respectively) and with microscopic evidence for the presence of cyanobacteria (violet). *Petrosia ficiformis* hosts the intracellular “*Ca*. Synechococcus feldmannii,” rather than the widespread “*Ca*. Synechococcus spongiarum” and is marked with two stars. Microscopic observations of cyanobacteria are based on the following references: (i) J. Vacelet and C. Donadey, J Exp Mar Biol Ecol 30:301–314, 1977; (ii) K. M. Usher, R. S. G. Toze, J. Fromont, J. Kuo, and D. C. Sutton, Symbiosis 36:183-192, 2004; (iii) K. M. Usher, J. Kuo, J. Fromont, S. Toze, and D. C. Sutton, Eur J Phycol 41:179–188, 2006), (iv) M. Pfannkuchen, S. Schlesinger, A. Fels, and F. Brummer, J Exp Mar Bio Ecol 390:169–178, 2010; (v) P. M. Erwin, S. Lopez-Legentil, and X. Turon, Microb Ecol 64:771–783, 2012; and (vi) I. Burgsdorf, P. M. Erwin, S. Lopez-Legentil, C. Cerrano, M. Haber, et al., Front Microbiol 5:529, 2014. The vertical bars represent the medians, the boxes represent the first to third quartiles, and the whiskers show the lowest or highest datum within 1.5 times the interquartile range of the lowest and upper quartile, respectively. The data points beyond the ends of the whiskers are outliers. Download FIG S1, PDF file, 0.5 MB. Copyright © 2019 Burgsdorf et al. 2019 Burgsdorf et al. This content is distributed under the terms of the Creative Commons Attribution 4.0 International license.

### The functional genomic repertoire of *Parasynechococcus*-like sponge-associated cyanobacteria.

Nine symbiotic cyanobacteria were compared to 19 *Parasynechococcus* cyanobacteria (Table 1; see Data Set S1, sheet 1, in the supplemental material). The choice of free-living *Parasynechococcus* species used for comparative genomics here is explained in (see Text S1 in the supplemental material). The genes of three “*Ca.* Synechococcus feldmannii” and six “*Ca.* Synechococcus spongiarum” cyanobacteria (Data Set S1, sheet 1) were assigned to 1,378 clusters of orthologous groups (COGs) and to 1,008 SEED functional roles. In comparison, the free-living genomes analyzed here received a higher number of COG and SEED annotations per genome (see Fig. S2 in the supplemental material). Agglomerative hierarchical clustering based on COG and SEED functional categories grouped the symbiont genomes together and apart from the closest free-living cyanobacteria. Symbiotic and free-living cyanobacteria were characterized by different enrichments of specific functional categories compared to free-living counterparts (see Fig. S3 in the supplemental material). A significantly lower proportion of functional categories in symbiotic genomes was confirmed by a lower number of genes with both COG and SEED annotations (Fig. S3 and Text S1). However, when considering both absolute counts and similar function based on COG annotation, only two SEED categories were truly enriched in symbiotic genomes: DNA metabolism and iron acquisition and metabolism (Fig. S3).

10.1128/mSystems.00057-19.1 TEXT S1 The supplementary text contains discussion related to (i) selection of relevant reference genomes for comparative genomics analysis of sponge-associated cyanobacteria, (ii) statistical enrichment analysis of genomic functional classes between sponge-associated and free-living bacteria, and (iii) relatively high numbers of COGs shared between “*Ca.* Synechococcus feldmannii” and free-living genomes. Download Text S1, PDF file, 0.09 MB. Copyright © 2019 Burgsdorf et al. 2019 Burgsdorf et al. This content is distributed under the terms of the Creative Commons Attribution 4.0 International license.

10.1128/mSystems.00057-19.3 FIG S2 Plots of SEED (A) and COG (B) functional categories in symbiotic cyanobacteria (representing here unculturable and less-studied organisms) versus free-living cyanobacteria (representing here culturable and better studied organisms). The *x* and *y* axes represent square-root-transformed absolute counts and relative abundances of symbiotic (red dots) versus free-living (black dots) cyanobacteria. Download FIG S2, PDF file, 0.4 MB. Copyright © 2019 Burgsdorf et al. 2019 Burgsdorf et al. This content is distributed under the terms of the Creative Commons Attribution 4.0 International license.

10.1128/mSystems.00057-19.4 FIG S3 Dendrogram of data hierarchically clustered using Manhattan distances and Ward’s linkage method. The heat map represents root 4th transformed relative abundances of functions of SEED (A) and COG (B) categories. Numbers represent the absolute counts of functions. “*Ca*. Synechococcus feldmannii” (green), “*Ca*. Synechococcus spongiarum” (red) and free-living cyanobacteria (blue) are compared. Categories that were significantly enriched and depleted among symbiotic genomes are marked with green and red, respectively. Relative abundances of all the significantly different categories were correlated with the absolute counts of genes. Significantly depleted COG categories are indicated as follows: B, chromatin structure and dynamics; Q, secondary metabolites; M, cell wall/membrane/envelope biogenesis; I, lipid transport and metabolism; T, signal transduction mechanisms; G, carbohydrate transport and metabolism; and S, function unknown. Download FIG S3, PDF file, 2.7 MB. Copyright © 2019 Burgsdorf et al. 2019 Burgsdorf et al. This content is distributed under the terms of the Creative Commons Attribution 4.0 International license.

10.1128/mSystems.00057-19.5 DATA SET S1 The data set contains nine tables, including (i) 25 reference genomes analyzed here, (ii) 21 COGs shared between genomes of “*Ca*. Synechococcus spongiarum” and “*Ca*. Synechococcus feldmannii,” (iii) 40 functions related to symbiotic and free-living properties of “*Ca*. Synechococcus feldmannii,” (iv) 99 COGs shared between “*Ca.* Synechococcus feldmannii” and its free-living counterparts, (v) 23 *psb* genes among the genomes analyzed here of symbiotic and free-living cyanobacteria, (vi) eight genes related to the spermidine synthesis and methionine salvage pathways among the genomes analyzed here of symbiotic and free-living cyanobacteria, (vii) 14 COGs that were exclusively found in “*Ca*. Synechococcus feldmannii,” (viii) 27 proteins contained FN3 domains annotated in symbiotic cyanobacteria, and (ix) 63 proteins contained FN3 domains annotated in cyanobacteria (not sponge associated) according to the global search. Download Data Set S1, XLSX file, 0.05 MB. Copyright © 2019 Burgsdorf et al. 2019 Burgsdorf et al. This content is distributed under the terms of the Creative Commons Attribution 4.0 International license.

**FIG 2:**
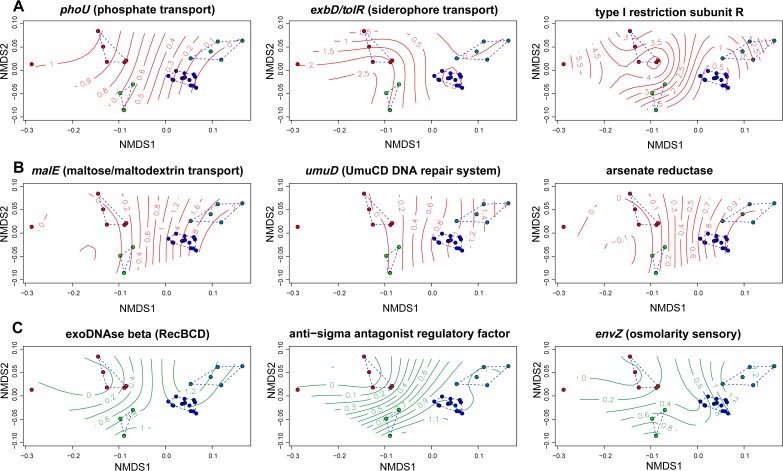
Nonmetric multidimensional scaling (NMDS) ordination plots on relative abundances of 1,430 SEED annotations of 28 cyanobacterial genomes, NMDS stress value = 0.058. Clusters of genomes produced are marked with a dashed blue line. Individual plots display overlaid smooth surfaces for significant (*P* < 0.01) representative functional roles (A) enriched in symbiotic cyanobacteria, (B) depleted in symbiotic cyanobacteria, and (C) common between “*Ca.* Synechococcus feldmannii” and free-living cyanobacteria. Numbers on the splines represent number of copies of the specific functional role. Red color splines represent distinctive features for symbiotic cyanobacteria. Green color splines represent common features for “*Ca.* Synechococcus feldmannii” and free-living cyanobacteria. “*Ca*. Synechococcus spongiarum,” “*Ca*. Synechococcus feldmannii,” *Parasynechococcus* clade, and *Parasynechococcus*/*Cyanobium* are marked with red, green, blue, and cyan dots, respectively.

Nonmetric multidimensional scaling (NMDS) based on 1,430 SEED functional categories produced four separate clusters: “*Ca.* Synechococcus feldmannii” genomes, “*Ca.* Synechococcus spongiarum,” *Parasynechococcus*, and *Parasynechococcus*-related *Cyanobium* (39) clades (Fig. 2). A total of 764 out of the 1,430 functional categories significantly correlated to NMDS coordinates (*P* ≤ 0.05). Symbiotic genomes were negatively loaded on axis 1 (Fig. 2A and B). Among the functions that influenced the separation between the two symbiont types, we detected a group of functional roles that appear related to the free-living stage of “*Ca.* Synechococcus feldmannii” (Fig. 2C). The dual lifestyle of “*Ca.* Synechococcus feldmannii” (free-living and resident within the host) is likely a source of a mixed gene composition in this symbiont, where some metabolic pathways showed similarity to the obligate symbiont “*Ca.* Synechococcus spongiarum” and others showed similarity to the closest free-living cyanobacteria, as detailed below.

### Sponge-specific features in the *Parasynechococcus*-like sponge symbionts.

Despite the overall functional similarity, “*Ca.* Synechococcus spongiarum” and “*Ca.* Synechococcus feldmannii” shared only 21 out of 1,378 COGs (Data Set S1, sheet 2). Shared depleted and enriched functions likely reflect their adaptation to a common niche: the host sponge. Among the shared depleted functions were genes related to maltose/maltodextrin and manganese transport, DNA repair system UmuCD, and arsenate reductase (Data Set S1, sheet 3). Among shared enriched functions, we found genes related to (i) siderophore-mediated iron transport and (ii) defense mechanisms against invading genomes.

**(i) Siderophore-mediated iron transport in sponges.** Symbiotic cyanobacteria were found to be enriched in genes related to the transport of siderophores: COG0609, COG1629, and COG4558 (Data Set S1, sheet 2). Eight out of nine symbiotic genomes contained genes related to siderophore transport, while the complete pathway of siderophore-mediated iron transport was found in one genome of “*Ca.* Synechococcus feldmannii” (277cV) and two “*Ca.* Synechococcus spongiarum” genomes (M9 and SP3). An operon including the iron regulation gene *irpA* (COG3487) was found in all genomes of “*Ca.* Synechococcus feldmannii.” Interestingly, COG3487 was mostly present in *Alphaproteobacteria* and *Gammaproteobacteria* (including the genera *Pseudomonas*, *Vibrio*, and *Agrobacterium*) and, according to eggNOG, was absent from all members of the *Parasynechococcus*/*Prochlorococcus* clade.

**FIG 3:**
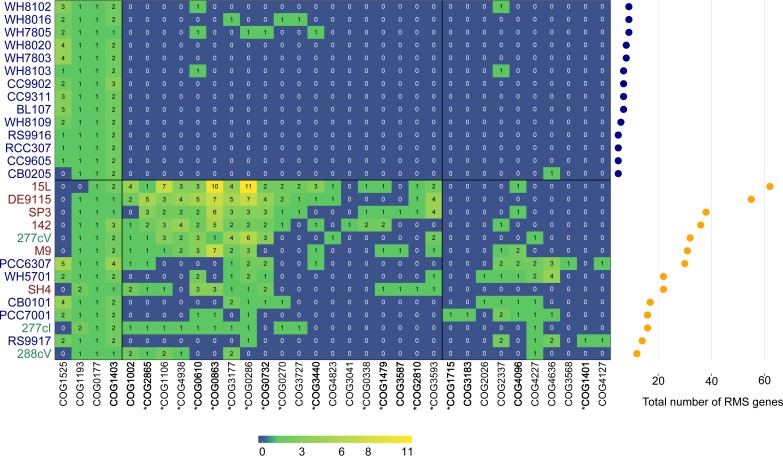
Heat map of absolute counts of genes from different COGs related to microbial defense systems related to “*Ca*. Synechococcus feldmannii” (green), “*Ca*. Synechococcus spongiarum” (red) and free-living bacteria (blue). The genomes were divided into two groups based on binary distance matrix. The total number of genes related to RMS is present on the right. All the genomes of “*Ca*. Synechococcus spongiarum” (red) are incapable to perform homologous recombination. COGs mentioned in Slaby et al. (77) as significantly enriched in genomes of sponge symbionts are marked with a star. COGs previously found as enriched in sponge-associated metagenomes in Horn et al. (76) are marked in bold.

**FIG 4:**
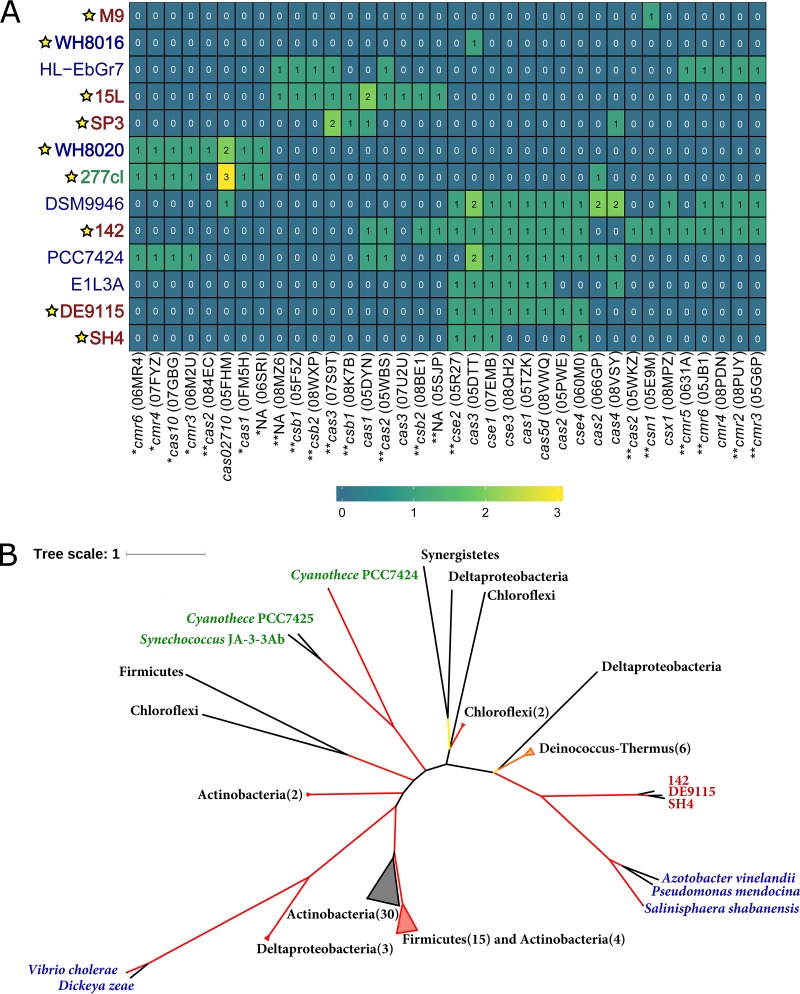
(A) Heat map of absolute counts of genes from different orthologous groups (NOGs) related to the CRISPR-Cas system. “*Ca*. Synechococcus feldmannii” (green), “*Ca*. Synechococcus spongiarum” (red), and free-living bacteria (blue) are compared. Only NOGs relevant for the genomes analyzed here (yellow star on the left) are shown. E1L3A represents Salinisphaera shabanensis E1L3A, HL-EbGr7 represents Thioalkalivibrio sulfidiphilus HL-EbGr7, PCC7424 represents *Cyanothece* sp. strain PCC 7424, and DSM9946 represents Meiothermus silvanus DSM9946. NOGs exclusively found in cyanobacteria and not found in cyanobacteria are marked with one star and two stars, respectively. (B) Maximum likelihood phylogenetic tree of the protein Cse1 (07EMB). “*Ca*. Synechococcus spongiarum” is marked in red, *Gammaproteobacteria* in blue, and free-living cyanobacteria in green. Branches with similar taxonomy were collapsed, and numbers of collapsed orthologs are shown in parentheses. The branches were colored according to the bootstrap values, ranging from yellow (55%) to red (100%).

**(ii) Defense mechanisms against invading genomes.** Out of the 21 common COGs, only COG1106, related to abortive bacterial infection (40), was present in all 9 genomes of sponge-associated cyanobacteria (Data Set S1, sheet 2), while among the free-living cyanobacteria, it was present only in cyanobacteria distantly related to the *Parasynechococcus*-like sponge symbionts (e.g., Trichodesmium erythraeum IMS101 and Nostoc punctiforme PCC73102). The total number and composition of restriction-modification system (RMS) genes in the symbiotic cyanobacteria, and also four members of the *Cyanobium* clade (e.g., *Parasynechococcus antarcticus*) and RS9917 (*Parasynechococcus equatorialis*), were higher and different, respectively, from those of the remaining 14 free-living cyanobacteria (Fig. 3). CRISPR-Cas is another type of defense mechanism against invading DNA. Eight and four CRISPR regions were found in the 277cV and 277cI “*Ca.* Synechococcus feldmannii” genomes, respectively. However, only 277cI contained a pronounced CRISPR-Cas-related region and harbored CRISPR-Cas-related genes (Fig. 4A). Genes related to CRISPR-Cas were also found in all six genomes of “*Ca.* Synechococcus spongiarum” and in 2 (WH8016 and WH8020) out of the 19 free-living *Parasynechococcus* clade genomes we analyzed (Fig. 4A). Surprisingly, 277cI shared seven CRISPR-Cas system eggNOG orthologs (NOGs) with the free-living WH8020 (Fig. 4A). In contrast, CRISPR-Cas-related genes found in “*Ca.* Synechococcus spongiarum” genomes resembled orthologs from other phyla or distantly related cyanobacteria (Fig. 4A). For example, *cse1* gene sequences found in three genomes of “*Ca.* Synechococcus spongiarum” were most closely related to orthologs from *Gammaproteobacteria* (Fig. 4B).

### Features potentially related to the free-living stage of “*Ca.* Synechococcus feldmannii.”

“*Ca.* Synechococcus feldmannii” is hypothesized to be a facultative symbiont (32, 33) and thus to have a free-living stage that may share relevant adaptive genes with the members of the *Parasynechococcus* clade. Indeed, we find that “*Ca.* Synechococcus feldmannii” shares 99 COGs with 19 free-living counterparts (Data Set S1, sheet 4). The shared genes were related to (i) cell surface and motility, (ii) DNA metabolism, (iii) environmental stress and cell regulation, and (iv) photosynthesis (Data Set S1, sheet 3). The relatively higher number of shared COGs between “*Ca.* Synechococcus feldmannii” and free-living *Parasynechococcus* compared to those shared between “*Ca.* Synechococcus feldmannii” and “*Ca.* Synechococcus spongiarum” is discussed in Text S1.

**(i) Cell surface and motility.** Enzymes involved in the synthesis of the O antigen residue l-rhamnose, including dTDP-glucose pyrophosphorylase (COG1209 [EC 2.7.7.24]), dTDP-4-dehydrorhamnose 3,5-epimerase (COG1898 [EC 5.1.3.13]), and dTDP-4-dehydrorhamnose reductase (COG1091 [EC 1.1.1.133]), were annotated in 17 out of 19 *Parasynechococcus* genomes and in “*Ca.* Synechococcus feldmannii” 288cV. dTDP-4-dehydrorhamnose reductase (COG1091 [EC 1.1.1.133]) was also annotated in 277cV. This is in contrast to “*Ca.* Synechococcus spongiarum,” in which these genes were found to be missing (36, 37), possibly to avoid host predation. Unlike “*Ca*. Synechococcus spongiarum,” all three “*Ca.* Synechococcus feldmannii” and *Parasynechococcus* genomes possessed a motion-related pilus retraction ATPase *pilT* gene (COG2805).

**(ii) DNA metabolism.** All three genomes of “*Ca.* Synechococcus feldmannii” harbored small and large subunits of exonuclease VII (or exodeoxyribonuclease VII). The *recBCD* pathway (exonuclease V), including alpha, beta, and gamma subunits, was present in the 277cV and 288cV genomes, while 277cI lacked the gamma subunit (COG1330). Both exonucleases were absent in “*Ca.* Synechococcus spongiarum” genomes and were present in all *Parasynechococcus* genomes (Data Set S1, sheet 3).

**(iii) Environmental stress and cell regulation.** The spermidine synthesis pathway (COG0421 [EC 2.5.1.16] and COG1586 [EC 4.1.1.50]), important for survivability under low temperatures and osmotic stress, was annotated in all three genomes of “*Ca.* Synechococcus feldmannii” and absent in “*Ca*. Synechococcus spongiarum” (Data Set S1, sheet 6). Anti-sigma and anti-sigma antagonist factors are adaptive gene expression regulators correlated to environmental changes (41). Anti-sigma antagonist (COG1366) and serine phosphatase RsbU, regulator of sigma subunit (COG2208), were among the 17 orthologous groups found present in all genomes of free-living cyanobacteria and lacking from “*Ca*. Synechococcus spongiarum” (141). Interestingly, eight of these orthologous groups, including COG1366 and COG2208, were shared between “*Ca*. Synechococcus feldmannii” and free-living members of the *Parasynechococcus* clade. Furthermore, “*Ca*. Synechococcus feldmannii,” like members of the free-living *Parasynecochoccus* analyzed here, harbored histidine kinase/phosphatase *envZ* and related transcription factor *ompR*, predictably lacking in “*Ca*. Synechococcus spongiarum” (Data Set S1, sheet 3).

**(iv) Photosynthesis.** Photosystem II *psbP* and *psbY* genes were absent in all six genomes of “*Ca*. Synechococcus spongiarum” and present in all three genomes of “*Ca*. Synechococcus feldmannii” (Data Set S1, sheet 5).

**Unique features of “*Ca.* Synechococcus feldmannii.”** The peculiar lifestyle of “*Ca.* Synechococcus feldmannii,” an intracellular symbiont that is horizontally transmitted, likely requires functions that differ from both its closest free-living relatives and from the extracellular, primarily vertically transmitted, symbiont “*Ca.* Synechococcus spongiarum.” Fourteen COGs were exclusively found in two out of three genomes of “*Ca.* Synechococcus feldmannii” (Data Set S1, sheet 7). FimT (COG4970), related to pilin structure, was annotated in all three “*Ca.* Synechococcus feldmannii” genomes (Data Set S1, sheet 3). Additional DELTA-BLAST analysis confirmed the annotation of the FimT domain in COG4970. Only two genomes from the *Parasynechococcus* clade that we analyzed harbored COG4970. All “*Ca.* Synechococcus spongiarum” genomes lacked these genes.

### ELPs.

**FIG 5:**
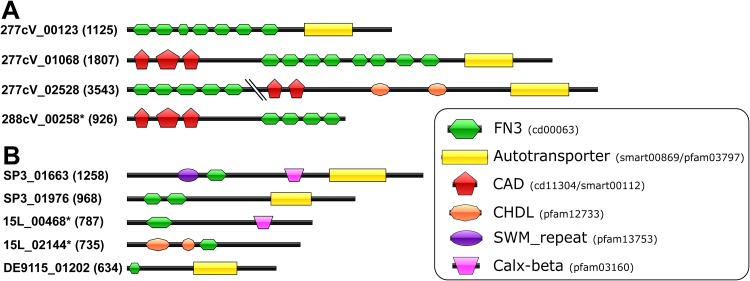
Domain architecture of FN3 proteins combined with additional types of domains derived from (A) “*Ca.* Synechococcus feldmannii” and (B) “*Ca.* Synechococcus spongiarum.” Names of proteins with an incomplete C terminal are marked with a star. The numbers of residues are written in parentheses.

Eukaryotic-like proteins (ELPs) found in the different symbiont types and free-living cyanobacteria are summarized in Table 2. The “*Ca.* Synechococcus feldmannii” 277cV genome contained 11 proteins with 80 fibronectin type III (FN3) domains (Table 2; Data Set S1, sheet 8). Three proteins (277cV_123, 277cV_1068, and 277cV_2528) showed a mixed-domain architecture (Fig. 5A) and also contained cadherin (CAD), cadherin-like (CHDL), and autotransporter domains (Fig. 5A). A search for proteins with a domain architecture similar to that of 277cV_123 in the Conserved Domain Architecture Retrieval Tool (CDART) database revealed 15 proteins: 14 of them belong the *Xanthomonadales* order of *Gammaproteobacteria*. Interestingly, one protein belonged to the cyanobacterial sponge symbiont “*Ca.* Synechococcus spongiarum” SP3: SP3_1976 (37) (Fig. 5B). For 277cV_1068, the proteins with the closest architecture belonged to the plant endosymbiotic nitrogen-fixing genus *Rhizobium.* The CHDL-containing protein 277cV_641 of “*Ca.* Synechococcus feldmannii” also harbored a CshA-type fibril repeat (CSHAF). Surprisingly, this protein architecture (CSHAF and CHDL) appears to be unique to “*Ca.* Synechococcus feldmannii.” CSHAF was also found in WH8102 (Parasynechococcus marenigrum) and WH7803 (Parasynechococcus pacificus); however, the domain composition of these proteins differed from 277cV.

**TABLE 2:** Summary of ELP types in symbiotic cyanobacteria

ELP	Predicted interaction(s)	Interaction with host cells/tissue	Result for ELP types:	
			“*Ca.* Synechococcus feldmannii” (*n* = 3)	“*Ca.* Synechococcus spongiarum” (*n* = 6)
Ankyrin	Avoidance of host predation	Archaeocyte	277cV	All genomes
LRR	Host specific	Unknown	All genomes	4 genomes (except 15L and M9)
TPR	Host specific	Unknown	Not enriched	Enriched in SP3
Rhamnose-free O antigen	Avoidance of host predation and phage resistance	Archaeocyte	277cV and 288cV	Absent
Cadherin	Adhesion to host cells	Unknown	Enriched in 277cV and 288cV	Enriched in DE9115
FN3^*a*^	Binding to host integrins and possible colonization	Extracellular matrix	277cV and 288cV	5 genomes (except M9)

a There were 3 to 11 FN3 proteins per genome (18 to 80 FN3 domains per genome) for “*Ca.* Synechococcus feldmannii” and 2 to 5 FN3 proteins per genome (2 to 12 FN3 domains per genome) for “*Ca.* Synechococcus spongiarum.”

Five out of six genomes of “*Ca.* Synechococcus spongiarum” also contained proteins with FN3 domains (Table 2). The “*Ca.* Synechococcus spongiarum” 15L, DE9115, and SP3 genomes had 5 proteins that contained other types of domains in addition to FN3 (Fig. 5 and Table 2). In contrast, the 19 free-living cyanobacteria analyzed here all lacked proteins containing FN3 domains (Table 2). A global search of proteins with FN3 domains within the entire phylum *Cyanobacteria* revealed 63 sequences that are not sponge associated; only 8 of them showed a mixed-domain architecture, like that found in the symbionts (Data Set S1, sheet 9). Among those 63 sequences, only one belonging to *Synechococcus* sp. strain GFB01 (out of more than 40 species of free-living cyanobacteria with FN3 domains) was found to be affiliated with clade 2 cyanobacteria (10), the same clade to which the *Parasynechococcus*-like sponge symbiotic cyanobacteria belong (Fig. 1). The two major taxonomic groups of free-living cyanobacteria that harbored FN3 domain proteins belonged to the *Nostocales* and *Oscillatoriales* orders (Data Set S1, sheet 9).

## DISCUSSION

### Different lifestyles result in different genomic adaptations.

**FIG 6:**
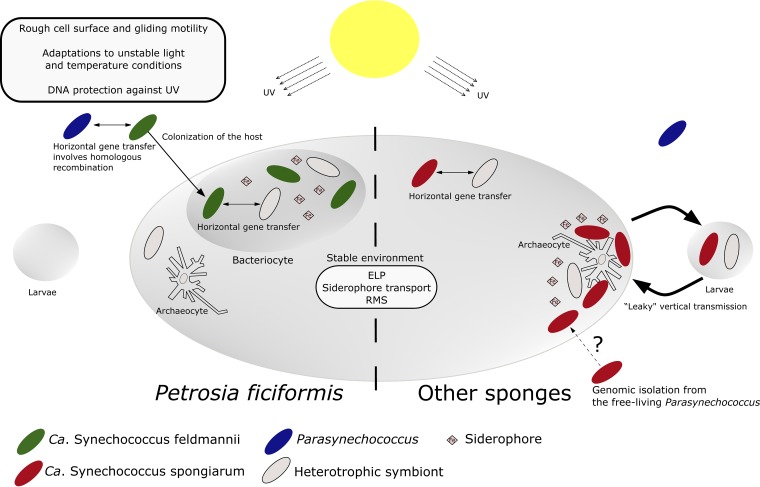
Schematic representation of genetic features that reflect different symbiotic lifestyles inherent to “*Ca*. Synechococcus feldmannii” and “*Ca*. Synechococcus spongiarum.” These differences incorporate types of symbiont transmission, degree of genomic isolation toward free-living cyanobacteria, iron metabolism, and cell surface properties related to interaction with the host archeocytes. RMS, restriction-modification systems; ELP, eukaryotic-like proteins.

The cyanobacterial sponge symbiont “*Ca.* Synechococcus feldmannii” is exceptional in terms of its unique association with the sponge species *P. ficiformis,* combined with its horizontal acquisition and intracellular location (Fig. 6). In contrast, a stable mutualism characterized by vertical transmission is inherent for the widespread cyanobacterial symbiont “*Ca.* Synechococcus spongiarum” (43). The unique lifestyle of “*Ca.* Synechococcus feldmannii” may reflect a more ancestral symbiotic relationship between sponges and cyanobacteria, before a stable mutualistic state was established.

“*Ca.* Synechococcus spongiarum” is vertically transmitted, so it likely does not need to survive a free-living state (outside the host). Contrarily, “*Ca.* Synechococcus feldmannii” is acquired from the environment at each sponge generation and thus must be able to survive a free-living stage and recolonize a sponge host. Furthermore, the extracellular “*Ca.* Synechococcus spongiarum” is exposed to sponge archaeocytes (sponge phagocytizing cells), while the intracellular “*Ca.* Synechococcus feldmannii” is protected from sponge archaeocytes by a physical barrier (the bacteriocyte membrane). We found that these differences in lifestyle are likely reflected in several genetic features of these two symbiont types (Fig. 6). We previously suggested that the typical O antigen of *Parasynechococcus*, composed of l-rhamnose (44), may be recognized by sponge archaeocytes as a signal for phagocytosis, while the lack of an l-rhamnose-based O antigen in the symbiont “*Ca.* Synechococcus spongiarum” (according to the missing biosynthetic pathway for this sugar) may enable this symbiont to avoid host predation (37). Free-living cyanobacteria, on the other hand, must maintain the l-rhamnose-based O antigen, in order to float in the water column (because O antigen mutants of free-living cyanobacteria sink [45]). As expected, the facultative symbiont “*Ca.* Synechococcus feldmannii,” which is predicted to have a free-living stage and thus a requirement to float, would need to maintain biosynthesis genes to produce the l-rhamnose-based O antigen. “*Ca.* Synechococcus feldmannii,” when found inside the host sponge, is suggested to use a different mechanism to protect itself from sponge archaeocyte phagocytosis: the compartmentalization inside bacteriocyte cells. We further speculate that “*Ca.* Synechococcus feldmannii” fails to colonize other sponge species, as in their mesohyl it will be vulnerable to host predation.

### Common adaptations to the sponge environment.

Iron is one of the vital factors for cyanobacterial metabolism due to its high requirements by the photosynthetic apparatus (46). Most marine cyanobacteria, including the *Parasynechococcus*/*Prochlorococcus* clade, lack production and transport of siderophores and rather use cell surface reduction of Fe(III) to the soluble Fe(II) (47). However, “*Ca.* Synechococcus feldmannii,” similar to “*Ca.* Synechococcus spongiarum” genomes, harbored a significantly higher number of genes related to iron acquisition, including siderophore transport. *Parasynechococcus*-like sponge symbionts lacked genes related to synthesis of siderophores and thus likely actively compete for siderophores from external sources (43). Discovery of the siderophore producer-consumer relations within the sponge-associated community will be an important step in understanding the network of chemical dependencies among the sponge community members. Interestingly, and dissimilar to “*Ca.* Synechococcus spongiarum,” “*Ca.* Synechococcus feldmannii” harbors an operon that includes an *irpA*-like iron-regulated gene (COG3487) that is related to iron starvation in *Synechococcus* sp. strain PCC7942 (48–50). Changes in environmental iron conditions may play a role for “*Ca.* Synechococcus feldmannii” to sense whether it is found inside or outside the host and accordingly trigger gene regulation related to either the symbiotic or the free-living state.

### ELPs: similarities and differences between the two symbiont types.

One type of common ELP is ANK repeat proteins. Pathogenic proteins containing ANK repeats were shown to prevent digestion of the pathogens in the vacuoles of the host immune system cells (51, 52). A similar mechanism is also used by nonpathogenic bacteria: for example, a very high number of ANK-containing proteins in insect-associated *Wolbachia* were linked to the interaction with the host (53, 54). FN3 domain proteins are another type of ELP linked to adhesion of the bacterium to host tissues. The adhesin protein FlpA (containing FN3 domains) was found in the pathogen Campylobacter jejuni and appeared to be essential for its attachment to epithelial host cells (55). Moreover, functional genomic and exoproteomic analyses of the S-layer-forming probiotic *Lactobacillus* revealed the presence of extracellular adhesins containing FN3 domains (56–58). ELPs such as ANK, CAD, FN3, and LRR domains were previously reported to be enriched in various (meta)genomic analyses of sponge symbionts (36, 37, 59–64), and also in the *Parasynechococcus*-like symbionts of sponges analyzed here. However, the different symbiotic properties of “*Ca.* Synechococcus feldmannii” and “*Ca*. Synechococcus spongiarum” likely influence the arsenal of ELP genes each harbor. The extracellular nature of “*Ca*. Synechococcus spongiarum” probably necessitates the observed higher diversity of genes with ANK repeats due to its direct exposure to the phagocytosing archaeocyte host cells (65, 66), while the requirement of “*Ca.* Synechococcus feldmannii” for sponge cell colonization explains the observed higher number of FN3 and CAD domains, which have adhesion properties. The enrichment of FN3 domains in sponge-associated bacteria was proposed to be involved in adhesion to the host cells by attachment to glycoprotein and structural proteins (60, 67), as well as cadherin domains, in adhesion to host cells and host colonization (60). Interestingly, we found that the expression of a sponge protein containing an FN3-like domain was positively correlated to the acquisition of “*Ca.* Synechococcus feldmannii” by *P. ficiformis* (M. Britstein, personal communication). FN3 domains with mixed-domain architecture from “*Ca.* Synechococcus feldmannii” showed higher similarity to *Alpha-* and *Gammaproteobacteria* than to free-living cyanobacteria and thus may have been acquired by horizontal gene transfer (HGT) from co-occurring sponge symbionts.

A high percentage of nonsynonymous mutations among ELP genes in “*Ca.* Synechococcus feldmannii” may reflect directional selection on these genes for improved colonization of the host. For example, adaptive mutations in adhesin genes of Escherichia coli increased binding to the polysaccharide structures of the host (68). Alternatively, the high mutation rates on genes related to host interaction may relate to their location on the cell membrane. Genomic islands characterized by higher mutation and HGT rates were found to contain genes involved in the formation of extracellular structures, among diverse free-living cyanobacteria, and their acquisition is generally thought to serve as a defense system against phage pressure (69–72). Interestingly, genes related to the CRISPR-Cas system in “*Ca.* Synechococcus feldmannii” also had a high mutation rate characteristic of genomic islands.

### Ecological isolation and genetic barriers in the two symbiont types.

The majority of the free-living *Parasynechococcus* strains analyzed here do not possess pronounced genomic barriers and are characterized by a large number of HGT events (73–75). Conversely, the symbiont genomes were found to be enriched in restriction-modification systems (RMSs) and genes related to abortive infection, in accordance with what has previously been reported for (meta)genomes of sponge symbiotic bacteria (59, 60, 76–79). RMSs (80, 81) and abortive infection systems (40) are efficient and widespread defense mechanisms against invading DNA in various bacteria. These systems promote genomic diversity by isolation of different genotypes from the genetic exchange with other lineages (80–83). The RMS (type I) allows bacteria to distinguish between self and foreign DNA according to methylation pattern. RMSs may thus promote HGT with distantly related bacteria of a similar methylation pattern, but may lead to genetic isolation toward closely related bacteria with different RMS repertoires (59, 60, 76). HGT can also occur by homologous recombination, involving *recBCD* (84, 85), which plays an important role in genome evolution of bacterial populations (86–89). RecBCD has multiple roles and can also function as a defense mechanism against invading DNA by degrading foreign DNA, if the crossover hot spot instigator (CHI) sequence is absent in the incoming DNA (80, 84). Earlier studies reported that a the presence of a high number of RMS genes in bacterial genomes correlates with the absence of *recBCD* (80), which is also the case in “*Ca.* Synechococcus spongiarum.” Thus, “*Ca.* Synechococcus spongiarum” may exchange genetic material only with other nearby symbionts that have similar RMS patterns, while it may have lost the ability for homologous recombination with other cyanobacteria (5). “*Ca.* Synechococcus feldmannii,” on the other hand, maintains the *recBCD* genes and has a free-living stage. It may, therefore, encounter other cyanobacteria and be capable of exchange of genetic information with the free-living *Parasynechococcus* strains by homologous recombination. Alternatively, homologous recombination involving RecBCD and ExoVII may also be linked to protection against UV damage (85, 90, 91) that “*Ca.* Synechococcus feldmannii” might face during the free-living stage. In accordance with the above results, and based on gene homology, we hypothesize that “*Ca.* Synechococcus feldmannii” obtained its CRISPR-Cas system through HGT from a free-living *Parasynechococcus* strain, while “*Ca.* Synechococcus spongiarum” probably acquired it from a sponge-associated gammaproteobacterial symbiont.

### “*Ca*. Synechococcus feldmannii”: special requirements to survive the free-living stage.

The facultative nature of the association of “*Ca.* Synechococcus feldmannii” with its host sponge requires the ability to adapt to changing environments and may involve more capacity for gene regulation than that of the obligate symbiont “*Ca*. Synechococcus spongiarum.” Despite a lower number of histidine kinases (COG0642) and DNA-binding response regulators (COG0745), “*Ca.* Synechococcus feldmannii” indeed differed from “*Ca.* Synechococcus spongiarum” in its repertoire of genes involved in genetic regulation and transcription, sharing with free-living cyanobacteria several components that were found lacking in “*Ca.* Synechococcus spongiarum”: for example, genes *envZ* and *ompR*, known to control the osmoregulation of Escherichia coli (42, 92).

While both symbiont types had a streamlined genetic component of the photosynthesis apparatus, the composition of these genes in “*Ca.* Synechococcus feldmannii” was more similar to that of free-living cyanobacteria and included *psbP* and *psbY* genes, which were lacking in “*Ca.* Synechococcus spongiarum.” These genes were probably lost in “*Ca.* Synechococcus spongiarum,” as it is always found in a more light-stable sponge environment and is characterized by lack of competition with different cyanobacterial species (37, 93, 94). However, such genes may be required in “*Ca.* Synechococcus feldmannii” to survive light shifts during the time it is found outside the sponge. Similarly, the presence of *N*-acetylmuramic acid 6-phosphate etherase (*murQ*), which is used in the reutilization of degradation products of peptidoglycan under limited-light conditions (95), may be important for “*Ca.* Synechococcus feldmannii” when the symbiont is found in seawater at low light levels. “*Ca.* Synechococcus spongiarum” lost this function. Moreover, spermidine is one of the most abundant polyamines in cyanobacteria (96, 97) and was previously linked to the replacement of damaged proteins under “chill-light” conditions (i.e., low temperature in combination with light) and osmotic stresses in *Synechocystis* sp. strain PCC6803 (98, 99). In Bacillus subtilis, the sigma-regulated phosphatase RsbU is involved in response to environmental stresses, including blue light and osmolytes (100, 101). The complete loss of genes involved in spermidine biosynthesis and sigma regulation genes in “*Ca.* Synechococcus spongiarum” may be related to a stable environment inside the host, while the presence of this biosynthetic pathway in “*Ca.* Synechococcus feldmannii” may be needed during the free-living stage.

Besides functional metabolic adaptations, “*Ca.* Synechococcus feldmannii” harbors cell surface elements that better resemble its free-living counterparts rather than the symbiont “*Ca.* Synechococcus spongiarum.” These include gliding motility-related *pilT* (102, 103) and pilus assembly-related *fimT* (104–106). The absence of these genes in “*Ca.* Synechococcus spongiarum” and their presence in “*Ca.* Synechococcus feldmannii” likely reflect the free-living stage of “*Ca.* Synechococcus feldmannii” and the need for host colonization, through increased contact with the host cells and motility toward the sponge.

In summary, we have shown that “*Ca.* Synechococcus feldmannii” combines features of planktonic and symbiotic picocyanobacteria. The facultative nature of its symbiosis with *P. ficiformis* would enable the host to select for the optimal symbionts according to the environmental conditions sensed. This would create competition among potential substrains of “*Ca.* Synechococcus feldmannii,” which are capable of exchanging genetic information with both open-ocean and symbiotic bacteria, promoting adaptive evolution.

## MATERIALS AND METHODS

### Sponge sampling, DNA isolation, and microbial DNA purification.

Two (no. 277 and 288) *Petrosia ficiformis* specimens were collected by SCUBA in January 2014 at depths of 27.3 and 14.9 m, respectively, from the Achziv nature marine reserve, Mediterranean Sea, Israel. Sponges were collected in compliance with permit 40246/2014 from the Israel Nature and National Parks Protection Authority. Only cortex tissue was used for further DNA extraction. DNA was extracted as described earlier (107). The microbial DNA fraction was enriched using New England Biolab's NEBNext microbiome DNA enrichment kit.

### Shotgun sequencing, assembly, and binning.

Genomic DNA was fragmented by sonication using Covaris S2 (Covaris, Woburn, MA). DNA libraries were prepared using the KAPA Hyper DNA library preparation kit with further Pippin Prep (Sage Scientific) size selection to 800 to 1,000 bp. Metagenomic shotgun libraries were sequenced on an Illumina NextSeq 500 platform (150-bp paired-end reads) in the DNA Services Facility at the University of Illinois at Chicago. Totals of 384.2 and 308.6 Gb of sequence for 277 and 288, respectively, were generated with mean insert sizes of 673 and 679.9 bp. Low-quality (minimum-quality threshold = 20) and short (minimum-length threshold = 50) reads and reads with ambiguous bases (“N”) were trimmed with the software sickle version 1.33, using a sliding-window approach (108). Sequence quality was evaluated using FastQC version 0.11.5 (109).

The 277cI genome was assembled *de novo* using IDBA-UD version 1.1.0 (110) (k-mer range = 40 to 80, step = 10). The 277cV and 288cV genomes were assembled from a subsampled version of the data set (10% of reads) using Velvet, with a k-mer size of 59, expected genome k-mer coverage of 41, and minimum cutoff of 15. Only scaffolds of ≥2 kb were used for genome binning. Genomes were first binned either based on visualization of a self-organizing map (ESOM) using tetranucleotide signatures as previously described (111) or on DNA fragment clustering via Barnes-Hut stochastic neighbor embedding (BH-SNE) in VizBin using pentanucleotide signatures (112). Second, assembly errors were rectified using REAPR version 1.0.18 (113). Third, completeness and contamination of the final bins were estimated with checkM version 1.0.7 (114). Then, protein sequences were obtained using Prodigal in metagenome mode (115). Finally, taxonomy affiliation of the predicted genes was obtained as detailed earlier (37).

### ORF prediction, domain search, and functional annotation.

Protein sequences were queried against the NCBI-CDD database using the Domain Enhanced Lookup Time Accelerated Basic Local Alignment Search Tool (Delta-BLAST, BLAST version 2.2.30+) and Reverse Position-Specific BLAST (RPSBLAST, BLAST version 2.2.30+), with E value cutoffs of 0.05 and 0.001, respectively. CAD and CHDL (cd11303, cd11304, PFAM00028, PFAM12733, and smart00736), FN3 (cd00063), and CSHAF (TIGR04225) domains were obtained from the Delta-BLAST results. The architecture of the relevant proteins was visualized with the IBS illustrator (116). COG annotation version 2014 (117) was assigned with Perl script cdd2cog.pl (https://github.com/aleimba/bac-genomics-scripts/tree/master/cdd2cog) using the RPSBLAST results. The amino acid sequences identified were also searched against the NCBI-NR database with DIAMOND (blastp, sensitive) and assigned to SEED/Subsystems annotation (118) using MEGAN 6.11.4. Additional functional annotation of protein sequences was performed by eggNOG-mapper and eggNOG database version 4.5 (119, 120), using the bactNOG data set and HMMER data mapping mode. The eggNOG website (http://eggnogdb.embl.de) was also used to obtain member lists of relevant orthologous groups. The Conserved Domain Architecture Retrieval Tool (CDART) (121) (https://www.ncbi.nlm.nih.gov/Structure/lexington/lexington.cgi) was used to obtain proteins with similar domain architecture. The Integrated Microbial Genomes (IMG) (122) (https://img.jgi.doe.gov/cgi-bin/mer/main.cgi) and Pfam (123) (http://pfam.xfam.org/) databases were used to obtain proteins with FN3 domains among cyanobacteria.

### Statistical analyses of the genome functional constituency.

Bray-Curtis dissimilarities were calculated based on the SEED/Subsystems annotations using the vegdist function (vegan package) in R version 3.4.1. Multivariate nonmetric multidimensional scaling (NMDS) was created using the metaMDS function (vegan package in R) (124, 125). Clusters of the genomes were created using hclust (ward.D2 agglomeration method) and a cut height of 0.32 by the cutree function. Smooth surfaces were fitted using the thin plate spline method in the ordisurf function (vegan package). Heat maps were created using the superheat package (126) (Manhattan or binary distances, ward.D2 agglomeration method for hierarchical clustering). The Wilcoxon test (wilcox.test in R, package stats) was used to determine significant differences in relative abundances of COG and SEED functional classes between sponge-associated and free-living cyanobacteria. *P* values were corrected for multiple testing using the Bonferroni correction (p.adjust function in R), and categories with corrected *P* values of <0.05 were considered significantly different.

### Comparative genomic and phylogenomic analyses.

The phylogenomic tree was constructed using EDGAR (127) based on the core genome as detailed by Burgsdorf et al. (37). The pangenomes of symbionts were obtained as the set of all genes in the relevant group of genomes (37). For these purposes, open reading frames (ORFs) in the three “*Ca*. Synechococcus feldmannii,” six “*Ca*. Synechococcus spongiarum,” and 15 free-living cyanobacteria were identified with the classic RAST algorithm (128, 129).

### CRISPR detection and analysis.

CRISPR arrays were predicted using CRISPRFinder (130). Only CRISPRs described as confirmed were used for the further analysis. CRISPR-associated proteins were annotated using eggNOG-mapper. Protein sequences of Cse1 (07EMB) were obtained from the eggNOG website and aligned using GUIDANCE2 (131–133) (http://guidance.tau.ac.il/ver2/) using the MAFFT algorithm and 100 iterations. Ambiguous regions were trimmed from the alignment by trimAl (134) using the gappyout method. A maximum likelihood tree of 320 positions was calculated by the Le_Gascuel_2008 model (135), with gamma-distributed rate variation (1.4908), and a proportion of invariant sites (0.4688%) was constructed with MEGA 7.0.2 (136). Phylogenetic robustness was inferred from 100 bootstrap replications. The resulting tree was redrawn and annotated using iTOL (137).

### Distribution and abundance of cyanobacteria in sponge versus environmental samples.

To determine the distribution and abundance of “*Ca*. Synechococcus spongiarum,” we used the SMP data set (6, 138). 16S rRNA sequences were subjected to blastn 2.2.30+ (139) (E value threshold = 0.005) against the OTU representatives of the amplicon sequencing data set contains sponge and environmental samples (6). The OTUs with sequence identity of ≥99% were used to determine the relative abundance among the SMP samples. The binomial (presence/absence) *P* values of the host enrichment analysis of “*Ca*. Synechococcus spongiarum” among various sponge species and environmental samples were calculated as the binomial cumulative probability of the absence of OTUs as described previously (6), excluding *P* values that were corrected for multiple testing using the p.adjust() R function using the false-discovery rate (FDR) correction. Samples with corrected *P* values of <0.1 were considered significantly enriched with “*Ca*. Synechococcus spongiarum.” Only sponge samples that contained the ectosome part and which were not collected from the dark caves were further used to test the enrichment of “*Ca*. Synechococcus spongiarum.”

The PAM fluorometry measurements for each sponge species were obtained from Steindler (38).

### SNP calling.

SSRG.pl (https://github.com/PombertLab/SNPs/blob/master/SSRG) was used to create a synthetic library of artificial reads from the genomes of “*Ca*. Synechococcus feldmannii.” Further mapping of the either synthetic or sequenced raw Illumina reads against the reference genomes was done accordingly: (i) get_SNPs_IB.pl (https://github.com/PombertLab/SNPs/blob/master/SSRG) and FreeBayes (140) were used to call the variants between the reads and the reference genome, and (ii) SNPs were annotated using SNPdat_v1.0.5.pl (https://github.com/agdoran/snpdat).

### Data availability.

Fourteen sequences of genes contained FN3 domains derived from the genomes of “*Ca*. Synechococcus feldmannii” were deposited in the NCBI GenBank database under accession no. MK422179 to MK422192. The draft genomes of the *Ca*. S. feldmannii 277cV, 277cI, and 288cV have been deposited under Biosample no. SAMN10755897, SAMN10755956, and SAMN10755957, respectively (Bioproject no. PRJNA515489).
