# Supplementary figures and images for: Life at Home and on the Roam: Genomic Adaptions Reflect the Dual Lifestyle of an Intracellular, Facultative Symbiont
Source: mSystems. 2019 May 7;4(4):e00057-19. doi: 10.1128/mSystems.00057-19 (PMC6506613; doi:10.1128/mSystems.00057-19)

Sponge species

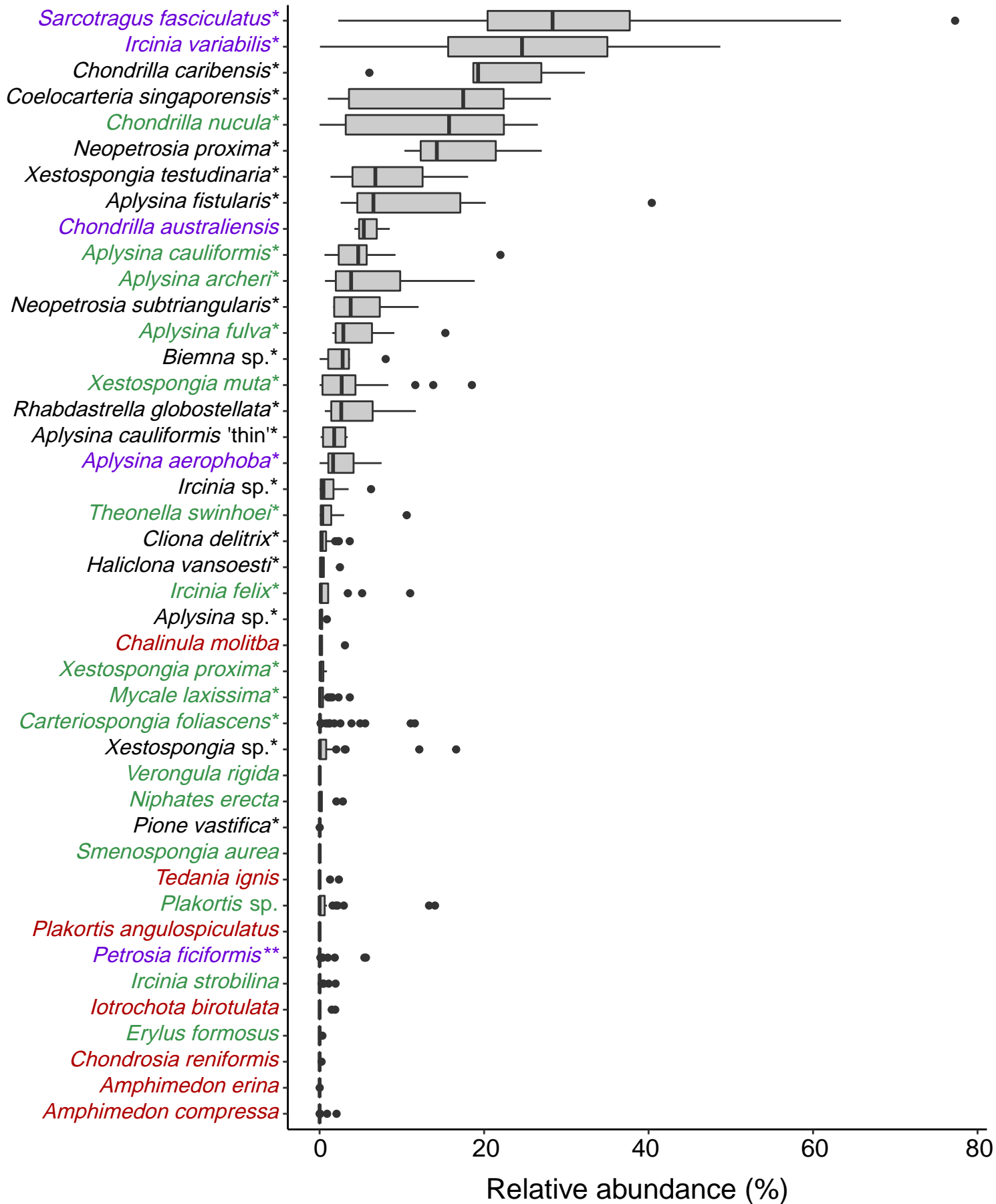

Supplement: FIG S1 [file mSystems.00057-19-sf001.pdf]

**A**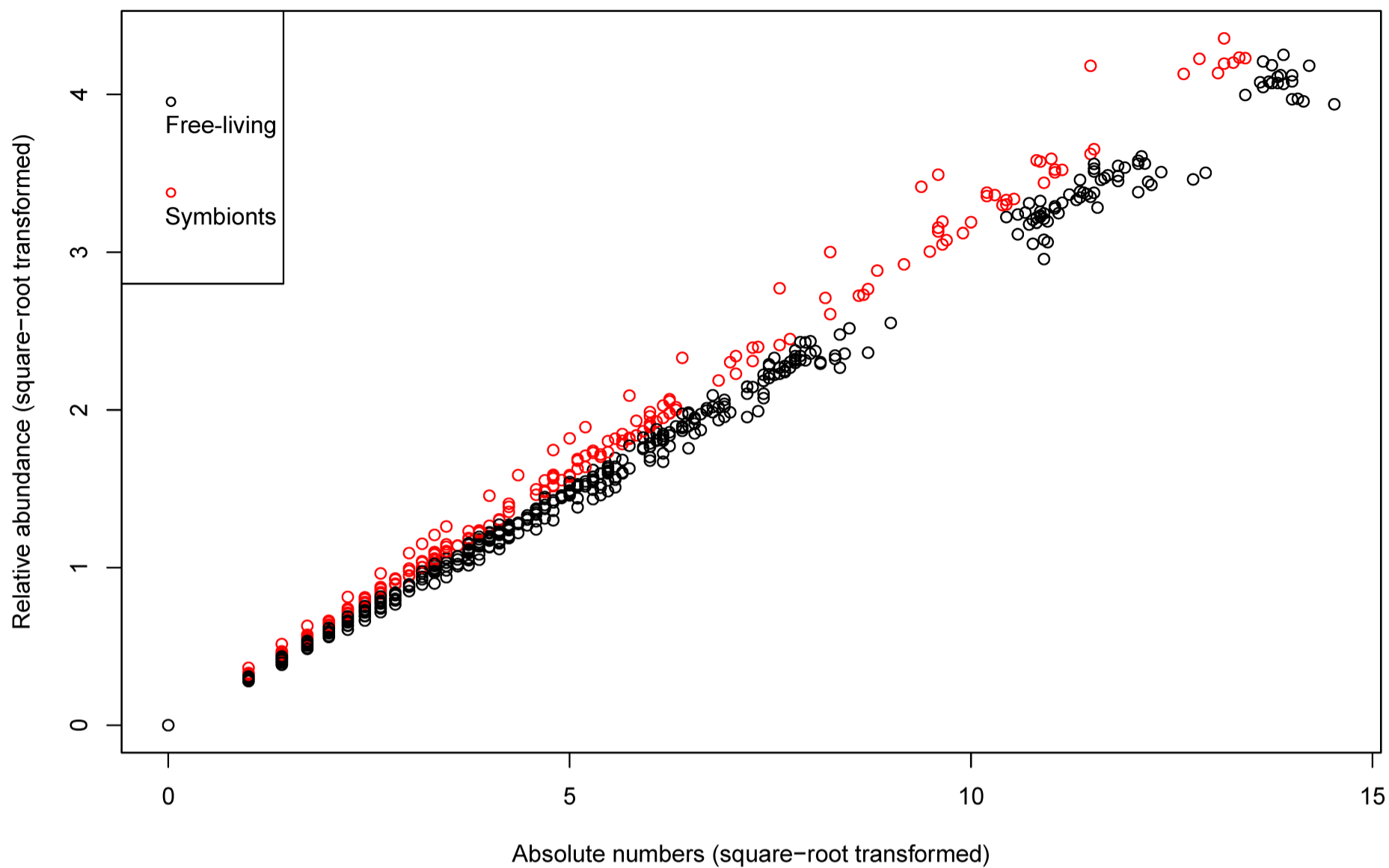**B**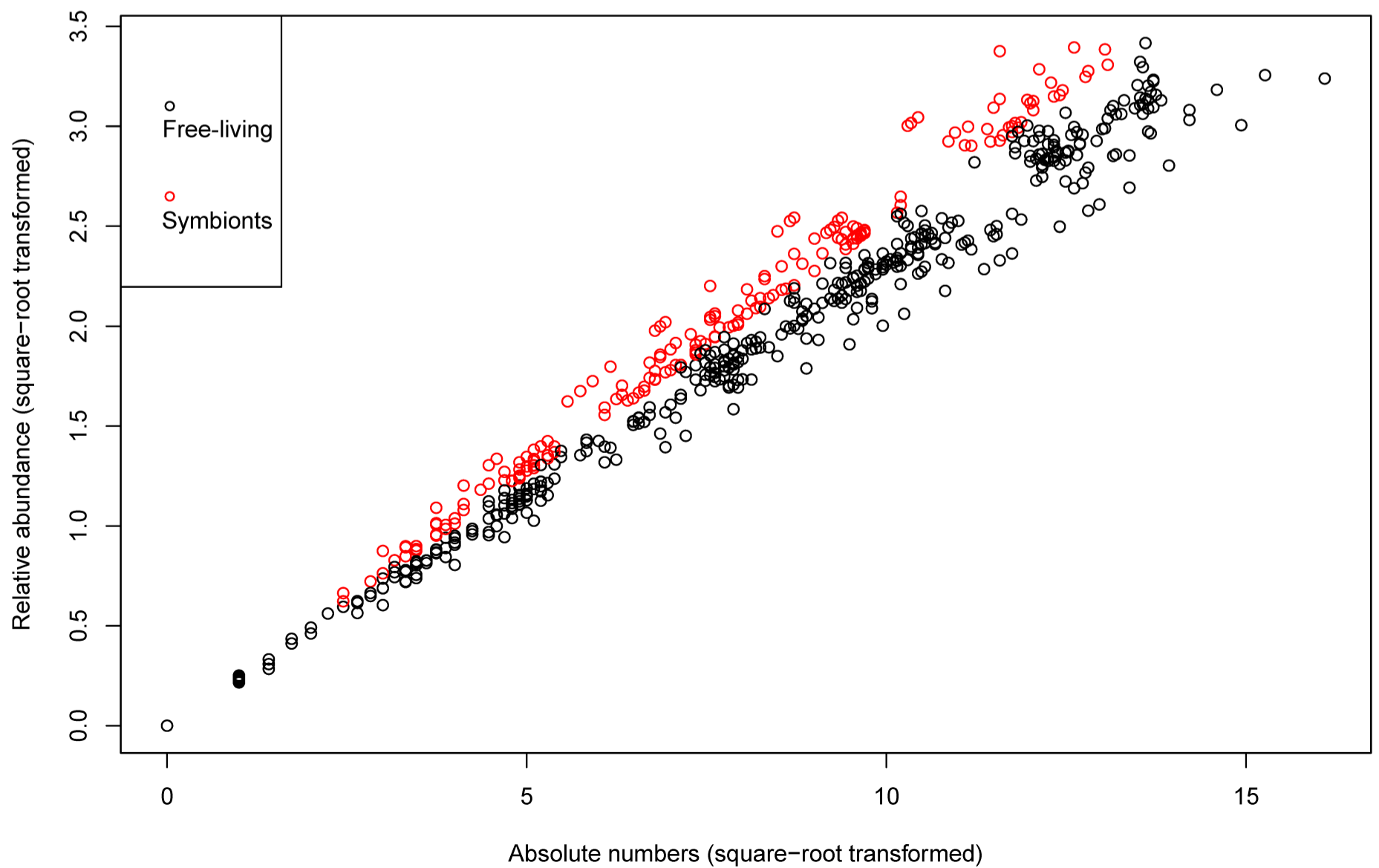

Supplement: FIG S2 [file mSystems.00057-19-sf002.pdf]
