# Supplementary material for: Life at Home and on the Roam: Genomic Adaptions Reflect the Dual Lifestyle of an Intracellular, Facultative Symbiont
Source: mSystems. 2019 May 7;4(4):e00057-19. doi: 10.1128/mSystems.00057-19 (PMC6506613; doi:10.1128/mSystems.00057-19)
Supplement: TEXT S1 [file mSystems.00057-19-s0001.pdf]

Methodological aspects: choice of genomes for comparison and absolute versus relative gene counts.

The impact of the choice of reference genomes used in comparative genomics was recently discussed in Díez-Vives et al., 2018 (141). In that work the list of the reference free-living cyanobacteria genomes compared to *Ca.* S. spongiarum also included distantly related basal lineages of cyanobacteria (*e.g. S.* PCC7336, *S.* JA-3-3Ab, *S.* JA-2-3Ba and *S.* PCC7502) (141). The evolution of such basal lineages was predicted to date approximately 1,300 Mya earlier than the evolution of marine planktonic picocyanobacteria (10-13). Besides evolutionary differences reflected by taxonomic affiliation, morphology (*e.g.*, single cell versus filamentous) and habitat, (*e.g.* marine versus acidic pools) are important factors influencing genomic content. Use of very different reference genomes could therefore confound the detection of symbiotic-associated functions. For example, the enrichment of ELPs among symbiotic cyanobacteria would not be detected, if distantly related cyanobacteria were included in the analysis. This may result from the potential role of ELPs in cell-cell or cell-substrate adhesion in filamentous cyanobacteria, while in the single-celled sponge-associated cyanobacteria ELPs are suggested to be involved in the interaction/colonization of the host. Therefore, if we include reference genomes from free-living bacteria that are evolutionary very distant, we may miss genomic features that are related to the symbiotic status of the studied bacterium. We are thus in agreement with Diez-Vives et al. on the fact that much thought needs to be given to the selection of genomes used in comparative genomics of symbionts. We further point out the importance of considering the evolutionary history of organisms included in analyses.

Additional consideration should be taken when using statistical enrichment analysis of genomic functional classes (*e.g.* SEED and COG). These analyses are often used for the comparison of the

relative number of genes in each category. However, such choice can result in a bias: as we compare the lower number of annotations per genome from less studied and mostly uncultured environments (such as the sponge microbiome) with the more completely annotated free-living counterparts. A very different scenario is obtained when, in addition to relative abundance, we compare also absolute numbers for each genomic functional class. The comparison of relative abundances resulted in 8 and 5 categories (SEED and COG respectively) being significantly higher in sponge cyanobacteria than free-living cyanobacteria, while when absolute counts and validation with additional annotation technique are considered, symbiotic genomes are truly enriched only in defense systems against foreign DNA (76) and iron acquisition categories. Relatively high number of COGs shared between *Ca.* S. feldmannii and free-living genomes (99 COGs) compared to those shared between the symbionts (21 COGs) may be also a result of the higher number of genomes and COG annotations per genome among the free-living *Parasynechococcus* (37, 141). However, eight out of 99 COGs shared between *Ca.* S. feldmannii and *Parasynechococcus* genomes were previously suggested as crucial for free-living cyanobacteria (141) and likely reflect the facultative nature of *Ca.* S. feldmannii.
