# Supplementary material for: Life at Home and on the Roam: Genomic Adaptions Reflect the Dual Lifestyle of an Intracellular, Facultative Symbiont
Source: mSystems. 2019 May 7;4(4):e00057-19. doi: 10.1128/mSystems.00057-19 (PMC6506613; doi:10.1128/mSystems.00057-19)
Supplement: FIG S3 [file mSystems.00057-19-sf003.pdf]

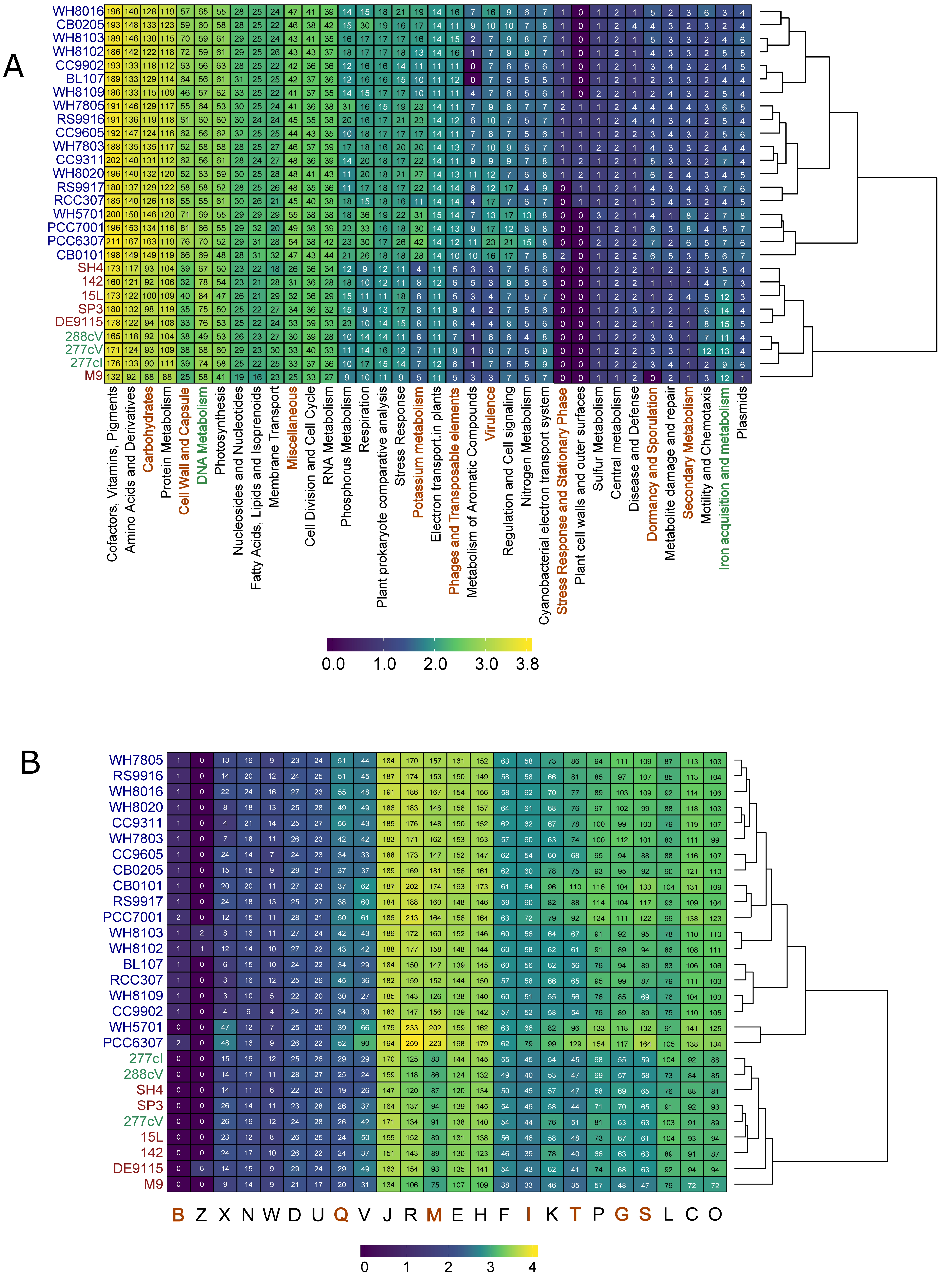
A
WH8016
CB0205
WH8103
WH8102
CC9902
BL107
WH8109
WH7805
RS9916
CC9605
WH7803
CC9311
WH8020
RS9917
RCC307
WH5701
PCC7001
PCC6307
CB0101
SH4
142
15L
SP3
DE9115
288cV
277cV
277cl
M9
Cofactors, Vitamins, Pigments
Amino Acids and Derivatives
Carbohydrates
Protein Metabolism
Cell Wall and Capsule
DNA Metabolism
Photosynthesis
Nucleosides and Nucleotides
Fatty Acids, Lipids and Isoprenoids
Membrane Transport
Miscellaneous
Cell Division and Cell Cycle
RNA Metabolism
Phosphorus Metabolism
Respiration
Plant prokaryote comparative analysis
Stress Response
Potassium metabolism
Electron transport.in plants
Phages and Transposable elements
Metabolism of Aromatic Compounds
Virulence
Regulation and Cell signaling
Nitrogen Metabolism
Cyanobacterial electron transport system
Stress Response and Stationary Phase
Plant cell walls and outer surfaces
Sulfur Metabolism
Central metabolism
Disease and Defense
Dormancy and Sporulation
Metabolite damage and repair
Secondary Metabolism
Motility and Chemotaxis
Iron acquisition and metabolism
Plasmids
0.0
1.0
2.0
3.0
3.8
B
WH7805
RS9916
WH8016
WH8020
CC9311
WH7803
CC9605
CB0205
CB0101
RS9917
PCC7001
WH8103
WH8102
BL107
RCC307
WH8109
CC9902
WH5701
PCC6307
277cl
288cV
SH4
SP3
277cV
15L
142
DE9115
M9
B Z X N W D U Q V J R M E H F I K T P G S L C O
0
1
2
3
4
